# Which Inter-Organisational Characteristics Supported More Effective Implementation of a New Zealand Falls and Fractures Prevention Programme? Applying and Adapting the Context and Capabilities for Integrated Care Framework

**DOI:** 10.5334/ijic.8924

**Published:** 2026-01-30

**Authors:** Maryam Pirouzi, Vanessa Selak, Tim Tenbensel

**Affiliations:** 1 School of Population Health, Faculty of Medical and Health Sciences, University of Auckland, Bldg. 507, Level 3, 22–30 Park Ave, Grafton, Auckland 1023, New Zealand; 2 School of Population Health, Faculty of Medical and Health Sciences, University of Auckland, Bldg. 507, Level 1, 22–30 Park Ave, Grafton, Auckland 1023, New Zealand

**Keywords:** integrated care programme, inter-organisational collaboration, falls and fracture prevention, organisational context

## Abstract

**Introduction:**

Why do integrated care programmes succeed in some settings but not others, even when national leadership and funding are aligned? This persistent question shaped our examination of the New Zealand Falls and Fracture Prevention Programme (FFPP), a complex, cross-sector initiative targeting older adults. We applied and extended the Context and Capabilities for Integrating Care (CCIC) framework to explore how organisational and inter-organisational factors contributed to variation in implementation and outcomes.

**Method:**

We conducted a qualitative comparative case study of four large districts with differences in FFPP implementation including 28 semi-structured interviews. Thematic analysis was primarily deductive, using the CCIC framework, but remained open to emergent, context-specific themes.

**Results:**

We identified 43 organisational and implementation factors, of which five had a particularly important effect on FFPP implementation and outcomes: a well-structured governance team, collaborative leadership, engagement with primary care and private organisations, positive prior collaboration experience, and applying a population-based approach. We modified the CCIC framework to more fully reflect our observations by adding prior collaboration experience and a life-cycle approach (from pre-engagement to establishment).

**Conclusion:**

The CCIC framework captured most key organisational dynamics but was enhanced by incorporating temporal and historical dimensions of collaboration.

## Introduction

The increasing demands posed by aging populations have highlighted the necessity for effective healthcare services, particularly for older individuals who are struggling with complex health needs [1,2]. Integrated care has emerged as a promising approach, offering potentially more efficient services for this demographic while maintaining costs akin to traditional care models [3,4,5]. While numerous integrated care programmes have been established internationally for people with complex health and social care needs, there remains a gap in understanding how to effectively implement and scale up such programmes within healthcare systems and in which contexts the programmes are most successful [6,7]. Various implementation theories and models offer insights into the contextual factors and organisational capabilities influencing the implementation of integrated care initiatives. These include the Rainbow Model for Integrated Care [8], which conceptualises integration across macro, meso, and micro levels; the Developmental Model of Integrated Care (DMIC) [9], which provides a staged model for developing integrated care; and the Comprehensive Theoretical of integration [10] which distinguishes structural, normative, and interpersonal dimensions of integration. While these models offer valuable conceptual foundations, they tend to emphasise system-level structures, care processes, or professional roles, with less focus on the organisational and inter-organisational capabilities required for implementation.

**Figure 1:**
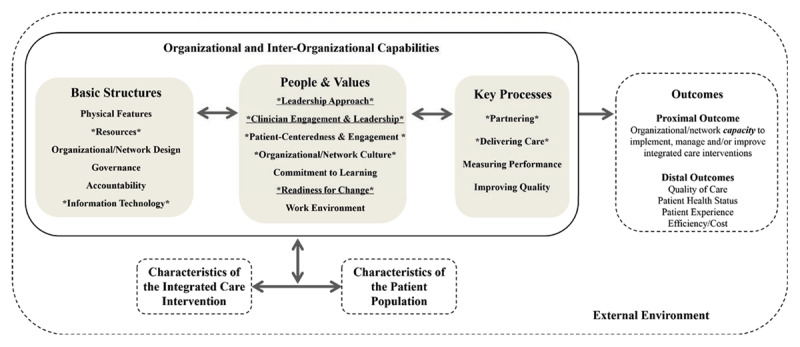
Context and Capabilities for Integrating Care Framework (Evans et al. 2017).

The Context and Capability for Integrated Care (CCIC) framework (Figure 1) addresses this gap by including multilevel contextual factors that determine the integration process. Developed through a comprehensive review of over 100 peer-reviewed articles and interviews with healthcare professionals, the CCIC framework conceptualises organisational context as a set of capabilities, that is, the coordinated structures, practices, and relationships that support integrated care delivery [11]. These capabilities span structural, social, and psychological dimensions, and are grouped into 17 factors across three domains: basic structures, people and values, and key processes [11,12]. While originally developed with a focus on organisational settings, the framework has since been applied to inter-organisational collaborations, including in this study, where integrated care required coordination across multiple entities and sectors.

To date, few empirical applications of the CCIC framework have been published. Asthana et al (2020) used this framework to investigate factors influencing the implementation and outcomes of a complex integrated care change programme in a local district in the UK and compared their results with Canadian case studies [13,14].

In this article, we used the CCIC framework as the basis for understanding why implementation and outcomes of a New Zealand integrated care initiative varied and which organisational and inter-organisational factors contributed to this variation. We selected the Falls and Fracture Prevention Programme (FFPP), a programme targeted at older adults comprising multi-component approaches and involving multi-sectoral collaboration among national and local organisations [15,16,17,18,19], as an exemplar of integrated care according to specific literature-based criteria (outlined below).

### Background on falls prevention and the FFPP in New Zealand

Similar to other high-income countries, falls are the leading cause of injury-related hospitalisations among adults aged 65 years and over in NZ [15,20]. An evaluation of NZ’s Injury Prevention Strategy in 2010 estimated the annual cost of falls was 18% of the total cost of injuries in the country [16].

**Figure 2:**
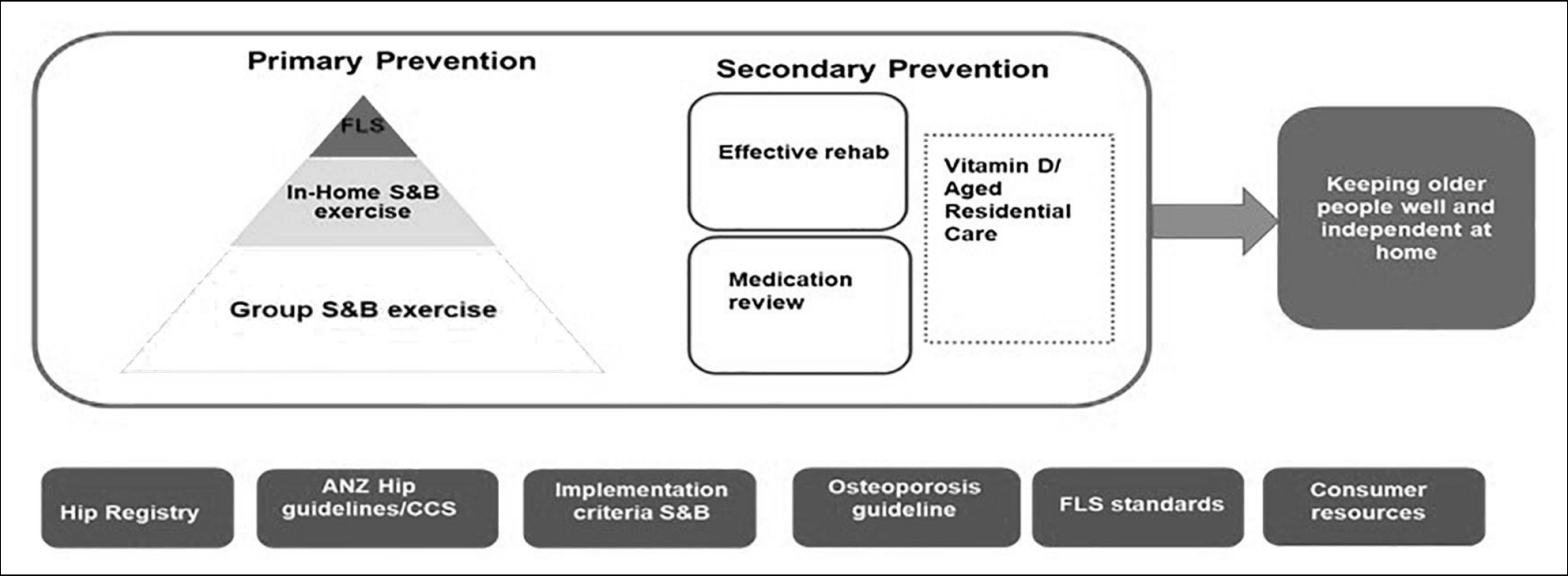
Falls and Fracture prevention program in New Zealand (Quality and Safety Commission,2020) [19].

In response to this issue, the NZ Government implemented the FFPP to reduce the incidence of falls and fall-related injuries in 2016. This programme targets falls among the elderly by using extensive primary (Community Strength and Balance (S&B) and In-Home S&B programmes and Fracture Liaison Service) and secondary prevention (effective rehabilitation, review medications and Vitamin D in residential aged care facilities) initiatives (See Figure 2). At an individual patient level, the FFPP coordinates services across community, or In-Home service, and secondary care providers based on whether the patient is at low or high risk of falls and fractures [19]. This focus on prevention is reinforced by cross-agency collaboration both nationally and locally. At the national level, government agencies such as Ministry of Health (MoH), Accident Compensation Corporation (ACC), and Health Quality and Safety Commission (HQSC) work together to coordinate the design, funding, and strategic oversight of the programme. Meanwhile, at the local level, a diverse array of organisations is involved in implementing various programme components. These include community-led agencies, District Health Boards (DHBs), which were government agencies responsible for managing hospitals and funding services locally, Primary Health Organisations (PHOs) comprising primary healthcare providers, private physiotherapy organisations, tertiary hospitals, and publicly funded non-government ambulance service providers (e.g., St John or other regional providers). This collaborative effort aims to deliver a comprehensive approach to falls and fracture prevention.

#### Selection of the FFPP as an exemplar of integrated care

The FFPP was selected as an exemplar of integrated care based on well-defined criteria drawn from the literature and tailored to the New Zealand context. The programme addresses a multifactorial health issue, falls and fracture risk, through a multi-component approach spanning prevention to rehabilitation. It targets older adults, a population with complex and high care needs. The FFPP demonstrates strong patient-centredness through its emphasis on tailored programmes such as risk assessment and individualised care planning. Critically, the programme is built on formal inter-organisational collaboration, supported by structured governance at both national and local levels, involving public, private, and NGO actors across community, primary, and secondary care. These features make FFPP not only an appropriate exemplar for studying integrated models in action, but also a strong candidate for applying the CCIC framework, which focuses on the organisational and inter-organisational capabilities required for successful implementation. A more detailed analysis of the programme’s operational design and district-level variation has been published elsewhere [21].

Although this programme operates nationally, its outcomes vary across regions [18]. For instance, in some districts, the number of new participants completing 10 or more community Strength and Balance sessions consistently exceeded national targets over multiple years. In others, despite having a similar number of class places available, referral volumes were lower and fewer participants reached completion thresholds. These disparities, evident in the HQSC Falls and Fracture outcome dashboards, highlight how differences in local governance, partnership structures, and implementation strategies can influence programme performance across sites [18].

These observed variations point to the importance of understanding how organisational and inter-organisational factors shape the implementation of complex integrated care programmes like the FFPP. To explore this, our research aimed to investigate the organisational and inter-organisational factors influencing FFPP implementation across four urban districts in New Zealand. In doing so, we also evaluated the adequacy of the CCIC framework for capturing and analysing these factors in a multi-organisational, system-level context.

## Methods

We applied a qualitative comparative case study design focused on the implementation of the FFPP in four large urban districts. These sites were selected to ensure variation in the organisational structures and delivery arrangements of the FFPP. All selected districts had large populations and substantial older adult demographics. By purposefully choosing sites with different inter-organisational configurations and governance approaches, the study aimed to explore how these contextual factors shaped the implementation and functioning of the same national programme in diverse settings. Recruited participants included senior managers, middle managers, programme coordinators (e.g., Community S&B and In-Home S&B coordinators), and working group members involved in FFPP implementation at the district level. Participants were drawn from all partner organisations to ensure comprehensive representation of organisational and professional perspectives. Selection criteria included relevant expertise, familiarity with the programme, and availability during the study period.

### Ethical approval for this study

Ethical approval for this research was obtained from the University of Auckland Human Participant Committee UAHPEC22004.

### Data collection and analysis

Between March 2021 and November 2021, semi-structured interviews were conducted with participants from each district including managers, clinical leads, coordinators, and planning and funding representatives. Participants were identified through consultation with national experts in falls and fracture prevention and public information sources; purposive recruitment ensured a balance of governance, clinical, and operational perspectives across all relevant organisations involved in FFPP implementation. All participants provided written informed consent before taking part in interviews, which were conducted either in person or via online platforms such as Zoom, depending on participant preference. The interview guide was informed by the CCIC framework’s domains (basic structures, people and values, and key processes) which include 17 organisational capability factors. We adapted the CCIC toolkit’s interview template to reflect the specific context of the FFPP in New Zealand. The final guide comprised three sections: (1) organisational structures and governance arrangements, (2) programme characteristics and implementation strategies, and (3) perceived outcomes and sustainability. The interview guide was piloted with two senior FFPP implementation stakeholders before finalisation. The full interview guide is available in Appendix 1. Recorded interviews were then transcribed with the assistance of an AI-generated transcription service (Otter.ai.) and all transcripts were edited to ensure their quality. NVivo 12 (QSR International Pty Ltd., Melbourne, Australia), a qualitative data analysis software, was used to manage qualitative data analysis due to the considerable amount of data. Transcripts were organised into separate files for each district, facilitating systematic data coding, grouping, and cross-site comparison.

Thematic analysis followed Braun and Clarke’s (2006) six-phase approach. First, the lead researcher (MP) familiarised herself with the transcripts through repeated reading and note-taking. We applied a primarily deductive coding strategy based on the CCIC framework’s 17 organisational capability factors, while remaining open to inductive codes where participants raised issues inadequately captured by the CCIC framework. To support comparative analysis, a coding matrix was created that listed each factor against participant roles (e.g., coordinators, funders, clinical leads) and sites, enabling systematic cross-case comparison.

As a final step, we interpreted how participants described changes in collaboration and implementation over time and synthesised these accounts into three broad stages of programme development: pre-engagement, service development, and business-as-usual. This stage-based view was not part of the formal coding structure but emerged as a useful lens for interpreting how organisational factors varied across phases.

## Results

We conducted 28 semi-structured interviews with managers and coordinators from partner organisations in the FFPP programme between March and November 2021, Table 1 shows the number of participants from each district and organisation. We identified 43 organisational and implementation factors and grouped them in five main categories which were primarily derived from the CCIC framework, We applied the three main CCIC framework categories: (1) basic structures, (2) people and values, and (3) key processes, and two specific factors related to the FFPP: 4) previous experience in the implementation of FFPP and 5) prevention approaches to FFPP.

**Table 1:** Participant recruitment summary.


LOCAL LEVEL	NUMBER OF INTERVIEWS			

	SITE A	SITE B	SITE C	SITE D

Community S&B Coordinator	1	1	1 (Joint)*	

In-Home S&B provider	1	1	1(Joint)	

Fracture Liaison Services	–	2	1	1

Planning and Funding Manager	1	1	1	2

Clinical leader	1	1	1	–

ACC regional Injury Prevention rep	1	1	–	–

PHO Coordinator	1	3	1	

St. John Pathway manager	1	–		1(Joint)

**Total**	**8**	**10**	**6**	**4**


*Site C and D had shared community and In-Home S&B programme.

To facilitate comparison and identifying important organisational and inter-organisational contextual factors, we classified districts as high, moderate, and low-performing based on three outcomes: community awareness of the programme, enhanced service delivery, and programme maturity. High-performing districts, such as Site A, were characterised by their ability to meet or exceed predetermined targets for the number of participants in all three components of the programme, had successfully sustained the programme and made it a business-as-usual initiative, and had a well-organised plan for raising awareness of the programme among older adults and professionals. In contrast, low-performing districts such as Site D struggled to reach participation targets, had not fully integrated the programme into their regular operations, and lacked a coherent strategy for promoting the programme, resulting in limited community and professional awareness and engagement.

Among the 43 organisational and inter-organisational factors and programme characteristics analysed, 22 varied substantially between districts. There was limited variation between districts in 11 factors, with another 10 similar across all four districts. Basic structure and key processes emerged as the two categories with a greater number of factors exhibiting variation between districts, as indicated in Table 2.

**Table 2:** Variation and similarities between districts in terms of organisational factors.


	VARIATIONS BETWEEN DISTRICTS		SIMILARITIES BETWEEN DISTRICTS	
	
	LARGE VARIATION	SMALL VARIATION	HIGH SIMILARITY	LOW SIMILARITY

**Basic structure**	**Staff**- having sufficient staff	**Funding**- reliance on ACC funding		Staff-Inter-professional connections

	**Staff**- innovations in service delivery more efficiently	**Funding**- using community and private sector capacity		

	**IT-** Quality of referrals between organisations	**IT**- E-referral between community- primary and secondary care.		

	**Governance and accountability**- well-structured working group			

	**Governance and accountability**- partnerships of all partner organisations			

	**Governance and accountability**- frequent working group meetings			

	**Governance and accountability**- clarity of partner organisations responsibility			

**People and Value**	**Clinical engagement**- clinical engagement in working groups	**Commitment to learning**- formal platform for sharing knowledge and experiences	**Attitude towards collaboration**	**Attitude towards collaboration**- primary care engagement

	**Clinical engagement**- clinical leadership of each component of programme	**Commitment to learning**- informal platform for sharing knowledge and experiences	**Common vision and values**- share same objectives and values/managing conflicts	

**Key processes**	**Care delivery**-FLS-specialist engagement	**Care delivery**-Community S&B-involving different ethnicities	**Care delivery**-FLS- adherence to clinical guideline	**Partnership**- Ambulance service engagement

	**Care delivery**-FLS-Easy access to Bone scan	**Measuring outcome**- using full capacity of outcome dashboard	**Care delivery**-Community S&B-adherence to clinical guideline	**Measuring outcomes**- Perception on outcome framework capacity

	**Care Delivery**-Community S&B-providing community training and awareness	**Measuring outcome**- outcome framework capacity in showing inter-organisational relationships	**Care delivery**-In-Home S&B- adherence to clinical guideline	

	**Care Delivery**-Community S&B-using community capacity in service delivery	**Measuring outcome**- existing internal feedback and monitoring mechanism	**Partnership**- Partnership experiences	

	**Care delivery**-In-Home S&B-using private organisation capacity in service delivery	**Measuring outcome**- existing external feedback and monitoring mechanism		

	**Care delivery**-In-Home S&B-good flow between In-Home and other parts of the programme	**Partnerships-** enough referrals from primary care		

	**Care delivery**-In-Home S&B-good flow between In-Home and other parts of the programme			

	**Partnerships**- primary care engagement			

	**Partnerships**- primary care engagement in working group and governance level			

	**Partnerships**- primary care funding for the partnership			

	**Partnerships**- enough referrals between partner organisations			

**Programme characteristics**	Having previous programme before national partnerships			Previous partnership experience

	Approach to the prevention			


### 1- Basic structure

In the CCIC framework, the basic structure domain includes resources, governance, accountability, information technology, and organisational design. Among these, the presence of a well-organised governance team with clear roles and responsibilities emerged as the most critical structural factor in this study. In the early stages of implementation, regular steering group meetings were essential for coordination and decision-making. Districts varied significantly in their governance arrangements. For example, Site A established a dedicated service-level alliance for falls prevention with clear direction over three years. Once established, this team merged into broader workstreams (e.g., community services, health for older people) to support programme continuity. In contrast, Sites C and D created a joint steering group but lacked strong leadership and role clarity. These limitations reduced engagement with partner organisations and weakened coordination across entities. Participants noted challenges in inter-organisational contracting, especially between DHBs and PHOs, where collaborative and contractual roles became blurred, complicated decision-making. This lack of clarity hindered alignment and created tension among stakeholders, impeding effective programme governance.

“Initially, the service level alliance, a separate one was to make sure we had all of those elements working really well and had a set work plan. It had set targets and objectives to meet and meet all of those. So now it becomes more business as usual and fits under another workstream (health for older people) where we keep an eye on it, but it’s not an individual bit of work. (Planning & funding manager, Site A)”

“I think the DHB values collaboration. But there’s so many people you have to contact, and we have many other issues and a tiny funding team. There’s a collaborative relationship, but there’s also contractual relationship, and we have to balance the two. And when you’re working with the PHOs, the relationship between DHBs and PHOs is very difficult, because it was never clearly laid down what is the role of the DHB and what is the role of PHOs, and they’re constantly fighting around roles. (Planning and funding, Site C&D)”

Furthermore, adequate allocation of staff and optimization of human resources are crucial factors for ensuring streamlined service delivery and enhancing the quality of patient care. Our assessment identified differences between districts: Site B faced a shortage of human resources for almost all components, while Site D lacked personnel specifically for the Falls Liaison Service (FLS). Conversely, Sites A and C had sufficient staffing to cover all three components of the program. Interestingly, while these districts allocated adequate human resources, not all chose to hire additional staff; instead, they leveraged available resources by empowering local private organisations. However, while having enough human resources was important for programme functionality and continuity, enabling the existing workforce, such as training health assistants for specific programme components, enhanced its sustainability.

“What they did very well here and was actually an idea of one of the physios in my clinic is that we need a physio or nurse to do the initial assessment and set it all up. Still, then the treatments after that are more like motivating them, which don’t need a lot of medical knowledge. So, they (F&FSLA team) have allowed us to use a physio assistant to do follow-ups. (falls champion, Site A)”

“I can see clearly that the FLS nurses have had a turnover, whether that’s due to the role itself. I don’t think so because they have been registered nurses that have stayed long term; they seem to enjoy that work. It’s more about job security and so I think if we can solve the job security issue, and that’s to do with the notice period of when the contract ends and getting DHB board decisions and ACC board decisions early and then that would help with that continuity issue. (ACC representative, Site B, C & D)”

Moreover, establishing an efficient e-referral system between community, primary, and secondary care providers was essential. This system facilitated seamless communication and coordination, strengthening inter-organisational relationships and ensured efficient patient transfers and information exchange. However, while there were e-referral systems between primary and secondary care providers in all districts, none of them achieved a fully mature approach to connecting community, primary, and secondary services. The challenges of establishing e-referral systems between organisations especially between private organisations, has also been highlighted by participants.

“Especially the electronic referral management system itself, has made a big difference to how easy it is to make all sorts of referrals. Once upon a time, we had to print off a long document off the pathways and then send somebody a fax and it was time consuming and cumbersome, and GPs were not that interested. For some obviously, that did the job, but it was a barrier. So, for all services, we’ve sought to make the process of referrals streamlined and simple. That’s improved referral rates considerably. (Primary care Physician, Site A)”

“We’re a separate entity, we come up against the challenge of security when passing on these referrals. So currently, we don’t have a secure system, we’re working on getting My Practice [an electronic practice management system] so that we can do e-referrals. (Ambulance rep, Site B)”

Interestingly, financial resources (as part of resources factor in CCIC) did not emerge as a primary influencing factor in FFPP implementation. This may link to similar financial support across four case study sites as all were large districts, removing it as a distinguishing factor. Instead, the study scrutinizes the complex dynamics between districts and external funding from ACC to discern their reliance and associated challenges. There were also diverse funding models applied to different components of the program. For example, in the community S&B program, contracts between the DHB and leading sports organisations funded coordinators rather than service delivery itself, resulting in positive responses from participants involved in the program. In Site B, General Practitioners received funding for screening individuals at risk of falling, and referring them to relevant programme components, illustrating a more proactive approach to primary care engagement.

“For the group funding, I was surprised at how well they did actually because I thought that in their model where they were just found a lead provider, they wouldn’t provide any money for the patient to the free. I was skeptical, but I was pretty pleasantly surprised that they got such large uptake. (Planning and funding, Site C&D)”

“So, [Site C and D], they’re always together, they always do things together as one, well, [Site B] is separate, the main obvious differences, for Site B we have this falls prevention contract where we (GPs) are funded to [identified people at risk] and refer them for falls prevention programmes. Well, in [Site C and D], we don’t have that set up yet, but they are looking into it and might implement the same programme for those DHBs if they see that it’s successful, but for [PHO] It’s definitely a successful programme. (Portfolio manager, PHO, Site B)”

### 2- People and values

According to the CCIC framework, the people and value category encompass seven key factors: leadership approach, clinical leadership and engagement, patient-centeredness and engagement, commitment to learning, organisational culture, work environment, and readiness to change. All but two of the seven CCIC factors were raised by participants. We observed variations between districts in leadership approach, clinical leadership, and commitment to learning, however, attitudes toward collaboration and common vision and values were similar and positive. Among these, collaborative leadership and clinical engagement were the most important factors in implementing falls prevention initiatives. Collaborative leadership focuses on building an environment that brings together all professionals and organisations and facilitates open conversations. We found that districts with collaborative leadership showed a higher level of clinical engagement in all programme components.

“For the change of the number of people who fall in the community, you need people who have skin in the game, those people who fall in the community, not those people who work in buildings and healthcare. People who have skin in the game so, you could take a metaphor of the more people from bureaucracy that are in a meeting, the less chance there will be an outcome. (Clinical lead, Site A)”

“Various members of the team are clinicians, so we’ve got (name of new clinical leader) now. So, he represents the geriatricians, we’ve got a clinical nurse who heads it. We’ve got allied staff. So, I think there are a lot of clinicians engaged in it. (Geriatrician, Site B)”

While all participating organisations showcased a supportive and positive attitude toward collaboration, instances of prior negative collaboration experiences with central government agencies, and the intermittent focus on falls prevention over two decades led to some skepticism among participants, particularly in Site B. Across all sites, organisations shared a common vision and goals centered around preventing falls among older individuals and supporting their independent living at home,. For example, the involvement of the largest ambulance service in the FFPP represents a significant shift in their operational focus. Traditionally focused on providing emergency acute care, St. John had embraced a role in proactive health prevention. This change underscores their enthusiasm and commitment to broadening their contribution within the healthcare system, aligning well with the shared objectives of the FFPP.

“Exciting that we could start doing this type of thing that we’ve moved away from being in the emergency service that we used to be known as, and we’re now like trying to do health prevention as well. (Regional representative, ambulance service, Site A)”

The commitment to learning and knowledge-sharing played a crucial role in establishing connections among providers within and across organisations or districts. However, its direct influence on improving outcomes was relatively limited, for instance in efforts in sites C and D to enhance connections among Community Strength and Balance providers through formal and informal sessions.

“We want to be able to offer benefits, so to the providers are a part of this network. So, we started doing our networking lunches, where we had a guest speaker (University academics) … One instructor that is their own business, they don’t have any affiliation with any organisation and that sort of thing. So, when they join our programme, they feel like they’re part of something bigger, so they can also network with like-minded people. (Community S&B coordinator, Site C&D)”

However, this did not result in increased engagement of older participants in classes or higher completion rates of strength and balance sessions, which represented a key indicator on the outcomes dashboard.

### 3- Key processes

Among factors in the key process category, the partnership process stands out as a fundamental element in the successful implementation of the FFPP. It required active involvement from primary care, community providers, and private organisations. Districts in which PHOs were engaged at the governance level and community providers were empowered, had better outcomes and a sustainable programme. For instance, site A demonstrated proactive involvement with primary care from the program’s outset, collaborating with local In-Home service providers to engage proactively with GPs in their regions and simplified risk assessments process. However, in the low performing district, (Site C) engagement with primary care providers at the governance level was not consistent.

“We very keen to be understood that the way in which primary care works is different from other organisations on the whole. And it’s always an opportunity to say this is how it works in primary care. It’s great to be included as a voice, and it’s the heart of the health system. (primary care physician, Site A)”

“I think our programme (site C) was criticised for not engaging primary care sufficiently. That was probably fair, in some ways, Site A was engaged more strongly in primary care. (Public health physician, Site C)”

Monitoring performance and establishing feedback mechanisms, both internally and externally, are vital for continual quality enhancement, especially in multifaceted programs involving multiple organisations. Despite quarterly reports collected from various entities, there was considerable room for improvement across all districts regarding the development of effective feedback mechanisms between organisations involved in different components of the programme as well as between national and local organisations. Moreover, all districts maintained similar patient flows for community and In-Home services based on clinical guidelines, ensuring consistent service quality across diverse districts.

“A particular challenge was receiving, getting referrals from general practice, and I think that was probably a failure of the model and the way we started the programme, we tried to engage widely and said the primary care was engaged, but they’re not engaged as one of the main partners, in terms of governance and setting up the model, and, for instance, [city name] engage general practice as the key partner, we didn’t do that. There are many reasons well; one of the primary reasons for not doing that was because Site A had one dominant PHO, Site D DHB had five at the time, and one PHO was quite dominant as well, which is [name of PHO]. (Planning and funding, Site C and D)”

### 4- Programme characteristics

The CCIC framework is designed to provide a general overview rather than a detailed exploration of specific programme or intervention characteristics, so the factors we presented here are specifically about the characteristics of programme. The findings of this study revealed that the most impactful characteristic of FFPP was districts adopting a population-based approach to prevention. This approach involved identifying patients at risk through comprehensive community training and awareness sessions, which significantly contributed to the programme’s success.

“So, we actually took a different approach to the other DHBs, because we strongly believed that it needs to be a population-based screening approach. And we also believed that if we really want to have an effect, for this intervention, we need to have large numbers of patients who need to be referred to the programme. So that is the reason we decided to go for this population-based programme. So, we involved all the six PHOs in this organisation here. And we had a contract with the PHO, in giving a certain amount of money for them to do this programme. (Clinical lead, Site B)”

Additionally, the existence of a pre-existing falls prevention program, rather than general experience with integrated care, was a crucial factor. For example, there was no nationwide falls prevention initiative in New Zealand for people over the age of 65, except for what was established in Site A in 2012. This locally driven programme represented a proactive response to the growing issue of falls among older adults. It involved early recognition of the problem, strategic planning, and implementation of targeted interventions. These efforts helped build local capacity and engage established private organisations in delivering services. This historical experience with falls-specific initiatives is distinct from broader collaboration history discussed in Section 5 and was instrumental in shaping Site A’s leadership and programme delivery approach.

“There was no falls prevention in New Zealand nationwide for people over the age of 65. Other than what we put in place in [Site A] in 2012, when we had decided that those people over the age of 75, were some of our most precious members of society, and the most at risk. And we had the strongest evidence for how you can change that, or some motivation or leadership are able to make that change. And I was fortunate enough to be in a position at a leadership table that I could make those issues more apparent to those people who could make financial decisions and strategic decisions.” (Clinical leader, site A)”

### 5- Additional factors beyond the CCIC Framework

In addition to the CCIC factors, participants emphasised the importance of previous collaboration experiences in integrated care initiatives. This factor, which reflects past collaboration experience (between organisations), significantly influenced the success or hindrance of new initiatives or partnerships. As expressed by a Community Strength and Balance coordinator from Site B, community groups experienced frustration with ACC-funded initiatives, particularly a strength and balance initiative through Tai Chi that was canceled despite initial enthusiasm. This history of funding withdrawal led to a sense of mistrust among community members, prompting questions about the longevity of new initiatives. Consequently, rebuilding trust emerged as a crucial aspect of fostering successful collaboration in the implementation of integrated care programmes.

*A lot of community groups complained about ACC initiatives and how they’re so great, and then they pulled the funding out, there was an ACC funded sort of balance strength and balance initiative through Tai Chi. I’ve heard about that, and it got cancelled, and people were gutted … there was a bit of mistrust when I started, like asking me, how long is this gonna last? So, I had to sort of rebuilding that trust. (Community S&B coordinator, Site B)*

While prior collaboration experience overlaps with the “People and Values” domain of the CCIC framework, we argue it functions as a cross-cutting contextual factor. It reflects the embedded history and inter-organisational memory that predate current collaboration, distinguishing it from emergent values or cultural traits. In our study, this was evident in the mistrust linked to earlier ACC-funded initiatives (Site B) and in contrasting histories of collaboration between DHBs and PHOs. For example, Site A’s long-standing relationships supported integration and programme uptake, whereas in other districts, unclear roles and strained DHB–PHO dynamics hindered collaboration. We therefore propose prior collaboration experience as a distinct factor shaping trust, engagement, and strategic alignment across CCIC domains.

### Modified CCIC Framework

**Figure 3:**
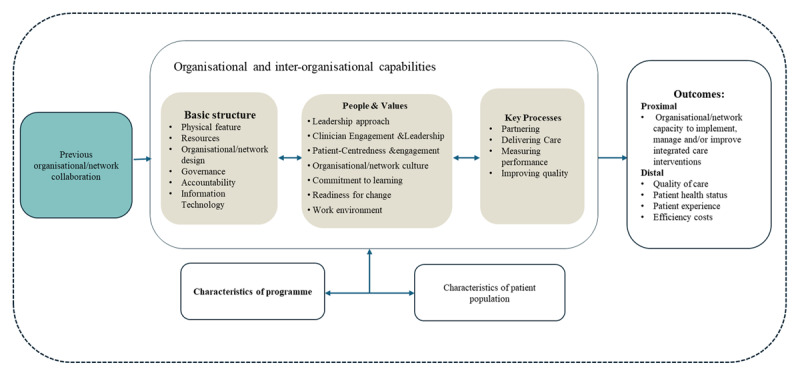
Modified CCIC Framework.

To capture this, we adapted the CCIC framework by adding prior collaboration experience (Figure 3). Historical relationships can strongly influence implementation by affecting trust-building, communication, and governance. Incorporating this dimension enables a more comprehensive understanding and management of integrated care initiatives, especially those involving multiple organisations.

### Life cycle approach in the implementation of integrated care

**Figure 4:**
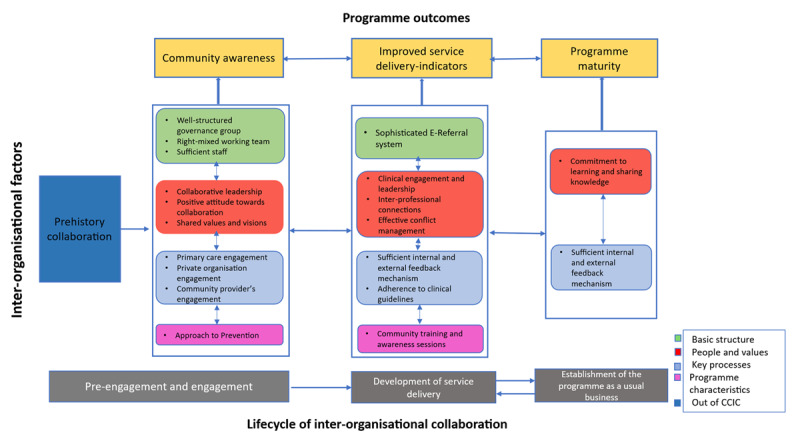
Important organisational factors in the implementation of the FFPP.

Through our thematic analysis, we identified shifts in districts’ focuses over time, this led us to determine three main stages in the implementation of the FFPP: (i) pre-engagement and engagement; (ii) development of service delivery; and (iii) establishment of the programme as a usual business. We identified the factors frequently mentioned by participants at various stages of the programme and their roles in facilitating collaboration. Then, we ordered factors based on their importance in different stages of the implementation (see Figure 4).

The initial stage involves introducing local partner organisations to the programme, setting up contracts, and establishing steering groups. Strong collaborative leadership and a well-structured governance team were important, along with the identification and engagement of key partners (primary care and private organisations) and adoption of a population-based prevention approach. The second stage focuses on developing service delivery, creating referral pathways, setting outcome targets, and resolving inter-organisational conflicts. Clinical engagement and effective conflict management were identified as crucial. In the third stage, the programme is established as a normal part of service delivery, and at this stage quality improvement, monitoring, and knowledge sharing become the most important factors. Effective monitoring and reporting systems facilitate continuous improvement and inter-organisational collaboration.

It’s important to highlight that the placement of key factors at various stages of programme implementation does not diminish their significance in other stages. Rather, it signifies that their role may be more prominent during that particular stage. By recognizing the significance of these factors and their alignment with the corresponding implementation stages, we were able to discern patterns, challenges, and areas of focus that are vital for achieving successful outcomes in integrated care interventions.

## Discussion

This study used the CCIC framework to examine variation in the implementation of New Zealand’s FFPP, selected as an exemplar of integrated care due to its complex, multi-agency structure. By comparing four districts with differing FFPP outcomes, we identified and categorised key organisational and inter-organisational factors across five domains, four from the CCIC framework and one additional factor.

Within the basic structure category, we found a well-structured governance team to be the most crucial factor, specifically where the programme involved multiple organisations. This aligns with the body of literature on integrated care initiatives [22,23,24]. For example, Gannon-Leary et al. highlights that well-structured governance promotes effective partnership, while Provan and Kenis identify essential governance dimensions such as shared goals, clear responsibilities, communication, and adaptability [25,26]. Our findings reinforce the pivotal role of governance, not only in its influence on other organisational factors within the context but also in its capacity to impact the desired outcomes within the programme.

Turning to “people and values”, collaborative leadership is identified as a fundamental factor that triggers other factors. Collaborative leadership plays a pivotal role in fostering commitments, building trust, and sustaining motivation among diverse organisations. This leadership approach encourages active participation from all partners, while the presence of well-designed governance teams establishes the necessary capacities for transparent and effective communication. Collaborative leadership was highlighted as crucial to the effective implementation of inter-organisational collaboration in numerous studies [27,28,29,30,31]. For example, in a study conducted by Auschra, a lack of leadership was identified as a hindrance that may cause uncertainties and hamper the further development of inter-organisational collaboration [31].

Huxham and Vangen identified four key dimensions of collaborative leadership: strategic direction, stakeholder involvement, cultural transformation, and relational dynamics. They argue that effective collaborative leaders need to be able to balance these dimensions and adapt their approach to the specific context of the collaboration [28]. Another study, by Klijn and Koppenjan, examined the role of leadership in collaborative governance, which involves the joint management of public issues by multiple organisations and stakeholders. They argue that effective collaborative leaders need to be able to create a shared understanding of the problem, build trust and commitment among stakeholders, and facilitate joint decision-making and action [32]. In addition, certain leadership characteristics such as openness to feedback and persuasiveness were observed as contextual factors supporting collaborative functioning [33].

This study extends the CCIC framework in two key ways. First, it identifies additional organisational factors-particularly prior collaboration experience, as influential in integrated care implementation. Second, it introduces a temporal model showing that specific capabilities become critical at different stages of implementation. While prior collaboration experience overlaps with the “People and Values” domain, we argue it functions as a cross-cutting contextual modifier. It reflects an embedded history of trust, disengagement, or mutual understanding that shapes current collaboration. Unlike emergent leadership or culture, this factor acts as institutional memory, influencing how quickly and effectively partnerships form. This aligns with Aunger et al.’s realist review, which highlights the role of historical interactions and reputations in building trust across organisations [34].

Our findings align with previous studies using the CCIC framework. Evans et al. 2016, highlighted organisational capabilities such as leadership, clinician engagement, and readiness for change [11], which were also prominent in our high-performing districts, although they did not formally prioritise these factors. Asthana et al. 2020, in applying the CCIC framework to integrated care systems in England, similarly identified governance and trust as foundational [13]. Our study supports these insights but contributes further by adding a temporal interpretation and identifying prior collaboration history as a key contextual factor.

Interestingly, we found less variation across sites in “People and Values” than in structural and procedural domains. This may reflect a shared national commitment to integrated care values in New Zealand’s health system. However, our analysis suggests that values alone are insufficient: how collaboration is enacted; through structures, processes, and leadership behaviours, has a greater impact on outcomes.

## Implications

These findings have practical implications for leaders of integrated care programmes. By identifying which organisational capabilities are most critical at different implementation stages, the study offers a roadmap to help health system leaders plan, sequence, and adapt interventions. The life-cycle perspective supports integrated care from initial engagement to sustained delivery.

Methodologically, the study used the CCIC toolkit in a qualitative capacity. Although the toolkit includes surveys and document analysis tools [12], its qualitative guidance is limited to identifying the three most and least important organisational factors. This constraint risks overlooking relevant contextual dynamics. To address this, our interview guide was expanded to include organisational factors, programme characteristics, and outcomes (Appendix 1). This adapted guide may assist other researchers conducting qualitative studies on integrated care.

The study also contributes to understanding how contextual factors vary in importance across different phases of collaboration. We identified three stages in programme implementation-pre-engagement, service development, and programme maturity-each marked by distinct enabling factors. While life-cycle models in integrated care exist (e.g. Minkman et al.’s four-phase model) [35,36] our study highlights how the significance of organisational capabilities shifts over time. These findings support the development of more effective, context-sensitive programmes and reinforce the need to account for evolving implementation challenges in policy and practice.

## Limitations

While the methodological approach used in this study- semi-structured interviews analysed through a structured framework, was well suited to the research aims- we acknowledge that a broader trimodal qualitative design could have added further depth. For example, incorporating observational methods, structural or documentary analysis, or the perspectives of patients and service users may have revealed different dimensions of implementation processes. Such approaches could illuminate further relational dynamics, workflow complexities, and experiential insights not readily accessible through interviews of programme coordinators and managers alone. Future research would benefit from integrating these complementary methods to expand on and deepen the organisational insights presented here.

## Conclusion

This study utilised the CCIC framework to examine the factors influencing the implementation and outcomes of the New Zealand Falls and Fractures Prevention Programme (FFPP). A comparative analysis of four districts revealed variations and similarities in organisational and inter-organisational factors, highlighting the most important elements in each CCIC category. Our findings enhance the CCIC framework by incorporating the additional contextual factor of “previous collaboration experience” and applying a life-cycle approach, emphasizing the importance of stage-specific interventions.

## Additional File

The additional file for this article can be found as follows:

10.5334/ijic.8924.s1 Appendix 1. Interview Questions.
