# Supplementary material for: Which Inter-Organisational Characteristics Supported More Effective Implementation of a New Zealand Falls and Fractures Prevention Programme? Applying and Adapting the Context and Capabilities for Integrated Care Framework
Source: Int J Integr Care. 2026 Jan 30;26(1):2. doi: 10.5334/ijic.8924 (PMC12857623; doi:10.5334/ijic.8924)
Supplement: Appendix 1. — Interview Questions. [file ijic-26-1-8924-s1.pdf]

Interview Questions

Part 1: Falls and fracture programme

1) **To begin, please tell me your discipline and role, in relation to the falls and fracture prevention programme. And how long have you worked as a part of Falls prevention programme**

2) Could you briefly describe the falls and fracture programme in your district?

Prompts:

- What does local model of care look like in your area and how are these aligned with the national framework?
-
- How long has this initiative or network been in place? Were there any pre-existing services before the falls and fracture programme?
- Who are your partner organisations at the district level and out of the district and what are their responsibilities? Do you think this partnership provided better service delivery for older people?
- Tell me about the process of patient screening and assessment for falls risk in your district?
- How the referral pathway works in your district (between community, primary and secondary system)
- To what extent is there greater integration and collaboration (at the local and regional levels)? And In which activity in falls and fracture programme, there is more integration between different providers?
- What internal or across-network challenges has your organisation faced in integrating care?
- What does your organisation do to integrate different organisation together?

3) How do you feel your organisation fits in with your partner organisations?

Prompts:

- Is there anything about your organisation that makes it harder to partner with other organisations?
- Is there anything about your organisation that makes it easier to partner with other organisations?
- How well do the partners in your integrated care initiative work together?

Part 2: Contextual Factors using the Context for Integrated Care Framework

1) Resources and capacity: financial and non-financial- information technology

- How this new model of funding works in your organisation? What have been the consequences of this partnership?
- Are the necessary systems in place to enable new ways of working?

- How does cross sector data sharing with different organisations occur within your district?

2) **Leadership style, governance, and accountability**

- Tell me about how you implement change in your organisation and how do you motivate your staff

- Who is represented/ are these right mixes of representation/ are they providing the right degree of oversight and direction/ how do you involve patients in this specific programme?

- How often are meeting of working group held?

3) **Organisational culture- readiness for change-work environment-commitment to learning- team work**

- What is your organisation attitude toward collaboration (resistant/ wary/open/ an advocate)?

- To what extend do you think organisations in your network have a common value and vision and goals/ strong sense of belonging to the new way of working

- Tell me about interprofessional teamwork at your network for falls and fracture programme

- Tell me about how your organisation learns from its past learning or from other external experts or other organisations experience and how shares experience with other districts

4) **Delivery of care- clinical engagement- patient -centeredness**

- What role does evidence (care pathway) play in how care is delivered/who can refer and how are people referred into services?

- Tell me about clinician engagement in governance and operation level

- To what extent do providers and patient engage in collaborative decision making

5) **Quality improvement and performance measurements**

- In there any plan in your district to improve the performance and quality of services of falls and fracture programme

Part 3: Outcome

- What benefits and outcomes (including shared outcomes) are being achieved, and how do these compare to those expected in the business case?

- What's working well, and not so well, and why?

- Can the new ways of working be replicated in other areas (such as investment into other populations, contexts, geographic areas, and conditions)? If so, how might this be achieved?

- To what extent do you think the outcomes framework and indicators represent a good relationship between different organisation?
